# Effects of fibroblast on electromechanical dynamics of human atrial tissue—insights from a 2D discrete element model

**DOI:** 10.3389/fphys.2022.938497

**Published:** 2022-07-26

**Authors:** Paul Brocklehurst, Henggui Zhang, Jianqiao Ye

**Affiliations:** ^1^ Engineering Department, Lancaster University, Lancaster, United Kingdom; ^2^ Biological Physics Group, School of Physics and Astronomy, University of Manchester, Manchester, United Kingdom

**Keywords:** fibroblast, cardiac model, discrete element method (DEM), electrical excitation, mechanical contraction, simulation, human atrial action potential model

## Abstract

Roughly 75% of normal myocardial tissue volume is comprised of myocytes, however, fibroblasts by number are the most predominant cells in cardiac tissue. Previous studies have shown distinctive differences in cellular electrophysiology and excitability between myocytes and fibroblasts. However, it is still unclear how the electrical coupling between the two and the increased population of fibroblasts affects the electromechanical dynamics of cardiac tissue. This paper focuses on investigating effects of fibroblast-myocyte electrical coupling (FMEC) and fibroblast population on atrial electrical conduction and mechanical contractility by using a two-dimensional Discrete Element Method (DEM) model of cardiac tissue that is different to finite element method (FEM). In the model, the electro-mechanics of atrial cells are modelled by a biophysically detailed model for atrial electrical action potentials and myofilament kinetics, and the atrial fibroblasts are modelled by an active model that considers four active membrane ionic channel currents. Our simulation results show that the FMEC impairs myocytes’ electrical action potential and mechanical contractibility, manifested by reduced upstroke velocity, amplitude and duration of action potentials, as well as cell length shortening. At the tissue level, the FMEC slows down the conduction of excitation waves, and reduces strain of the tissue produced during a contraction course. These findings provide new insights into understandings of how FMEC impairs cardiac electrical and mechanical dynamics of the heart.

## 1 Introduction

Roughly 75% of normal myocardial tissue volume is comprised of myocytes (Vliegen et al., 1991), which play the primary role in conducting cardiac electrical and mechanical activities in the human heart. However, fibroblasts are by number the most predominant cell type (Goldsmith et al., 2004), and while smaller in size than myocytes, they comprise 60%–70% of the total cell number in an adult mammalian heart (Baudino et al., 2006). In a normal cardiac tissue, every myocyte is closely neighboured and possibly coupled electrotonically by at least one fibroblast (Camelliti et al., 2005). Fibroblasts are believed to have profound impacts on cardiac function. Primarily, fibroblasts are responsible for the synthesis and maintenance of the extra-cellular matrix that surrounds and supports cardiomyocytes (Maleckar et al., 2009a). They also contribute profoundly to the structural, biochemical, mechanical, and electrical properties of the myocardium (Camelliti et al., 2005).

In various conditions, the population of fibroblasts increases with the progression of cardiac diseases. For example, fibrosis resulting from a large number of cardiac fibroblasts increases with aging (Camelliti et al., 2004; Camelliti et al., 2005), mitral valve disease (Corradi et al., 2005) or congestive heart failure (Li et al., 1999). In other cardiac diseases involving myocardial remodelling, such as cardiac hypertrophy, heart failure or infarction, fibroblasts play a central role in modulating cardiac dynamical behaviours (Camelliti et al., 2005).

There is growing evidence suggesting an increased incidence of atrial fibrillation with increased fibrosis (Everett and Olgin, 2007; Jacquemet and Henriquez, 2008). It has been hypothesized that excess connective tissue comprised of fibroblasts may act as a passive barrier to electrical impulse conduction (Kohl et al., 2005), causing conduction block or formation of re-entry (Xie et al., 2009a). In general, fibroblasts may affect electrophysiological properties of cardiac tissue (Camelliti et al., 2005), and hence affect its rate-dependent activities and promote formation of instable spiral wave excitations, facilitating atrial fibrillation (Zhan et al., 2014).

Computer modelling provides an alternative platform to experimental studies for investigating the functional impacts of fibroblasts on cardiac dynamics. In previous studies, computer models incorporating fibroblast-myocyte electrical coupling (FMEC) at various scales have been developed. These models include cell-pair coupling of myocytes and fibroblasts (Kohl et al., 1994; MacCannell et al., 2007), and a single myocyte coupling to one or more fibroblasts in the human atria (Maleckar et al., 2009a). Using these models, consequences of fibroblast-myocytes coupling on the action potential of myocytes have been further studied (Maleckar et al., 2008; Sachse et al., 2008). At the tissue level, effects of FMEC on cardiac excitation wave conduction in a one-dimensional strand of connected cells (Vasquez et al., 2004; Jacquemet, 2006) or in a cardiac bidomain model (Sachse et al., 2009) were also investigated. In addition, the effects of FMEC on cardiac excitation (Tanaka et al., 2007; Jacquemet and Henriquez, 2008) and arrhythmogenesis have been investigated using two-dimensional models (Zlochiver et al., 2008; Xie et al., 2009a).

There is considerable debate in the cardiac-related literature regarding whether fibroblasts contribute to cardiac electrophysiology in a passive or active manner. Traditionally fibroblasts were viewed as only passive obstacles or insulators (Kohl et al., 2005; Xie et al., 2009a). However, there is recent evidence that fibroblasts show homo- and heterogeneous cell coupling, allowing them to play some functional roles (Camelliti et al., 2005). It has been shown that fibroblasts formed functional gap junctions *in vitro* (Rook et al., 1992; Gaudesius et al., 2003; Chilton et al., 2007). It has also been shown that fibroblasts are able to conduct electrical signals over extensive distances with fibroblast-fibroblast coupling alone (Gaudesius et al., 2003). Cultured cardiac myocytes and fibroblasts readily form functional gap junctions (Rook et al., 1989; Camelliti et al., 2005). Evidence of gap junctional FMEC *in vivo* is sparser, though several studies have demonstrated its existence (Camelliti et al., 2004; Miragoli et al., 2007; Walker et al., 2007). However, the contribution of active FMEC on myocardial electrophysiology *in vivo* is still unknown, difficult to assess and controversial (Jacquemet and Henriquez, 2008; Xie et al., 2009a; Sachse et al., 2009). However, a recent review based on the most recent experimental evidence argued favourably the existence of fibroblast-myocyte electrotonic coupling (though with many more questions raised), calling for more studies to assess the impact of FMEC (Kohl and Gourdie, 2014).

So far, it is unclear how the FMEC affects cardiac electromechanical dynamics, especially with the myocytes being coupled to active fibroblasts instead of passive ones used in previous studies (Xie et al., 2009a; Zhan et al., 2014). Hence, the present study aims to investigate the effect of active FMEC in a two-dimensional human atrial tissue mode by considering active fibroblasts (Maleckar et al., 2009a), which may better represent the fibroblast action potential (Maleckar et al., 2009a; Kohl and Gourdie, 2014).

## 2 Methods

In the present study, the active fibroblast model developed in (Maleckar et al., 2009a) is coupled to an electromechanical myocyte model of the human atrial cell developed in our previous studies (Brocklehurst et al., 2015; Brocklehurst et al., 2017). This tissue model uses the Discrete Element Method (DEM) rather than the finite element method (FEM) to reflect the discrete nature of cell-to-cell arrangement in cardiac tissues. Using the 2D DEM model, we simulate and analyse the effect of FMEC on the electrical and mechanical activities of the atrial tissue for different intercellular coupling strengths and fibroblast populations.

### 2.1 Single-cell equations

#### 2.1.1 Myocyte model

The myocyte model used in this paper was introduced in (Courtemanche et al., 1998; Colman et al., 2013). It is a set of nonlinear differential equations describing the electrical action potential and mechanical kinetics of an isolated myocyte.

For the electrical action potential, we used the model of Colman et al. (2013) of human atrial cells, which was further updated by Brocklehurst et al. (2015), Brocklehurst et al. (2017) for simulation of atrial electro-mechanics (2015, 2017). The basal model is based on the Courtemanche et al. (1998) model, with updated formulations of ionic currents (Maleckar et al., 2009c), and a modification to the intracellular calcium handling system (Koivumäki et al., 2011) that were based on extant experimental data. In this paper we used the parameters of the model representing cells in the right atrial region as implemented in our previous studies.

For the mechanical model, we used the Rice et al. (2008) model for myofilament kinetics. The original Rice et al. (2008) model was adjusted to replicate the force-calcium relationship observed in human atrial cells, which was described and validated in (Brocklehurst et al., 2015; Brocklehurst et al., 2017). Electro-mechanical coupling was achieved by coupling the calcium transient produced by the single cell electrophysiological model into the updated Rice et al. (2008) myofilament model. In addition, a stretch-activated current was added to the membrane ionic current (*I*_*m*_), to model mechano-electric feedback (Brocklehurst et al., 2015; Brocklehurst et al., 2017). The model recreated the force-calcium relationship in human atrial myocytes and outputs a predicted cell shortening time course during the action potential, matching the experimental data (Brocklehurst et al., 2015; Brocklehurst et al., 2017).

#### 2.1.2 Fibroblast model

For the fibroblast electrophysiology, we used the “active 2” model developed by Maleckar et al. (2009a). The model consists of a set of differential equations to simulate the fibroblast membrane potential *V*_*f*_ and the dynamical changes of intracellular concentrations of *K* ^+^ and *Na* ^+^. The model was chosen as it can reproduce electrical activity of fibroblasts and, unlike other models, accounts for active membrane properties of fibroblasts, which are important to be represented in the FMEC model of the cardiac tissue (Maleckar et al., 2009a).

### 2.2 DEM representation of tissue mechanics

We used DEM to model the mechanical behaviour of the tissue, following the method introduced in (Brocklehurst et al., 2015; Brocklehurst et al., 2017). DEM is able to capture the intrinsic discrete nature of cardiac tissues. Unlike the traditional continuum-based methods, such as FEM, DEM allows us to study the effect of possible complex cell distribution, arrangement and distortion to fibre orientation on cardiac electro-mechanical dynamics.

As introduced in (Cundall, 1988; Hart et al., 1988), DEM tracks the dynamic interaction of circular rigid “particles.” Each particle’s position and velocity are tracked throughout the simulation, which is solved by an explicit time-stepping algorithm. A solid material may be modelled using DEM by bonding particles together using contact models, and a force-displacement law is solved at the contacts. Newton’s second law is used to determine the motion of each particle arising from the contact and body forces upon it. A brief outline of DEM application to human atrial tissue is presented here, and a full description of the method can be found in (Brocklehurst et al., 2015; Brocklehurst et al., 2017).

#### 2.2.1 Individual cell model

**FIGURE 1:**
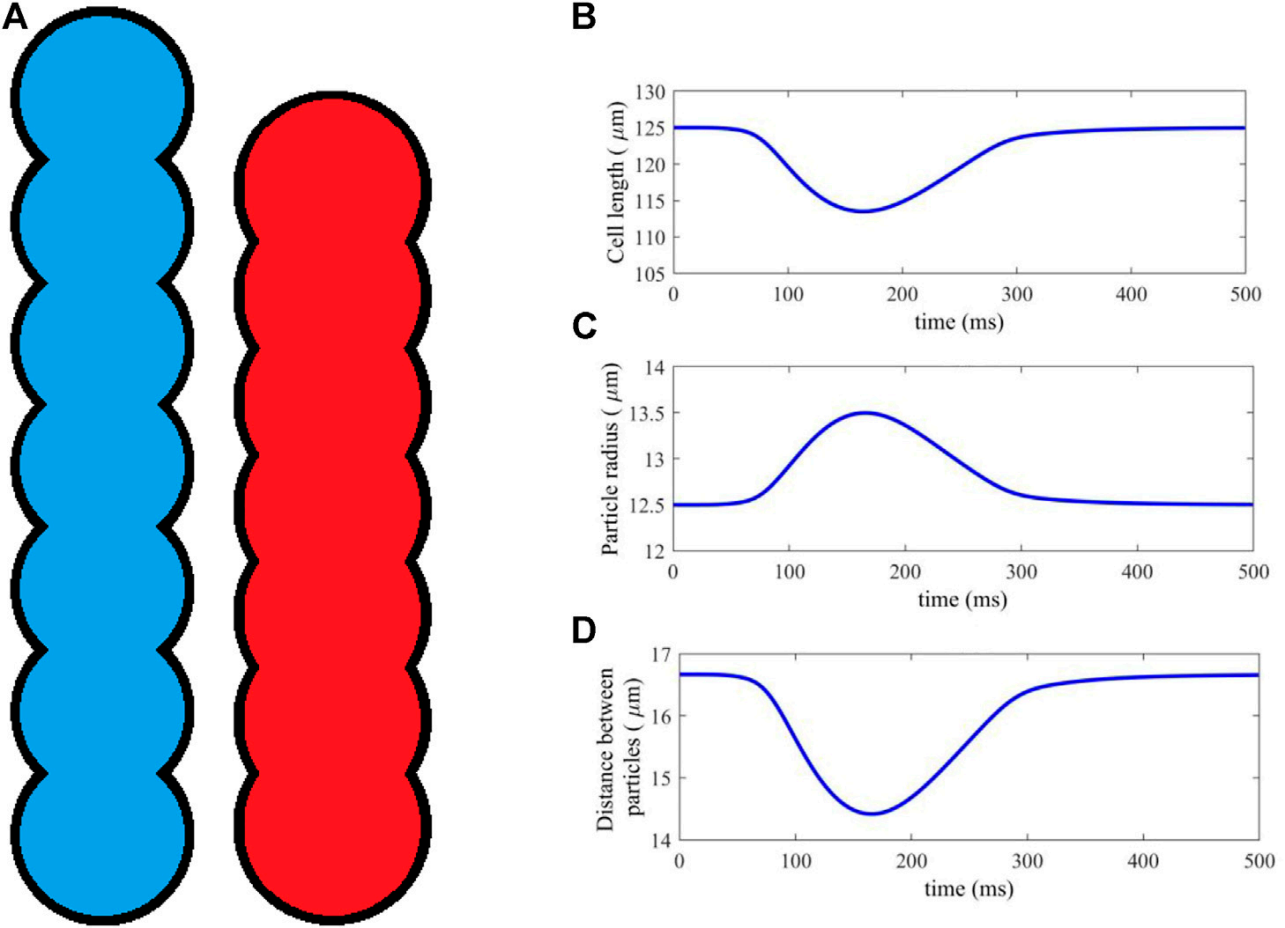
**(A)** An idealised geometry of a myocyte represented by 7 DEM particles, in its resting (blue) and fully activated contraction (red) states. **(B–D)** Cell length, particle radius and distance between particles, plotted against time for a typical time course of cell activation and contraction.

We choose a myocyte dimensions to be 125 μm in length and 25 μm in width, whilst fibroblasts have length 25 μm and width 25 μm similar to the study of Xie et al. (2009). DEM provides the option to group particles together into a “clump,” where the contact forces within the clump are ignored and the clump behaves as one cohesive object. Each myocyte in our model is represented by a clump of particles approximating realistic cell dimensions as shown in Figure 1.

Clumps are non-deformable, and so a method was introduced in (Brocklehurst et al., 2015; Brocklehurst et al., 2017) to simulate cell contraction by moving the particles along the cell axis, shortening its length. Atrial cells are an incompressible material, so that, in order to satisfy conservation of 2D area, the particle radii are scaled proportionately as the distance between them is altered, according to a simple geometric relation (Brocklehurst et al., 2015; Brocklehurst et al., 2017). Figure 1A shows the cell in its initial and fully deformed state, both of which have identical area. In this study, myocytes are represented by seven particles, each of which has a resting radius *r* = 12.5 μm and distance between adjacent particle centres *d* = 16.67 μm, which is close to the diameter of cardiac cell.

The length scaling of each myocyte is computed at each timestep of the simulation, using the sarcomere length output of the electromechanical single-cell model (Section 2.1.1). The sarcomere length is scaled to give the total myocyte length. An example contraction is shown in Figures 1B–D, showing the dynamical changes in cell length, particle radius and distance between particles against time during a full course of excitation and contraction. The result is a dynamic DEM clump that realistically contracts along its length according to the electrical and mechanical activities within the cell, and has constant cell surface area.

Fibroblasts in the current study are represented not by a clump, but by either a single particle or a group of non-clumped particles (see Section 2.2.2). The mechanics of fibroblasts are not well known, and to the best of our knowledge there is no mathematical model describing fibroblast contraction (Zhan et al., 2014). Hence in this study fibroblast-particles are assumed to be rigid and non-dynamic due to their significantly smaller size as compared to myocytes, with constant radius throughout the simulations.

#### 2.2.2 Tissue model

DEM contact bonds were formed between particles in the model. Following (Brocklehurst et al., 2015; Brocklehurst et al., 2017), we used a linear contact bond model, with an elastic spring of prescribed strength and stiffness in the normal and shear directions, as well as a dashpot in each direction to provide damping. Physically, these contact bonds represent connective material between individual cells, including the cell-binding protein structures desmosomes and other connective tissue. Contact bond parameters were chosen to capture the qualitative behaviour of these materials, which is primarily to prevent the separation and overlap of cells, and facilitate force transmission between cells. Full details and equations for the contact model and DEM formulation can be found in (Brocklehurst et al., 2015; Brocklehurst et al., 2017).

To model the interaction of fibroblasts and myocytes in cardiac tissue, previous studies have considered various methods of coupling the different cell types (Sachse et al., 2008; Zlochiver et al., 2008; Xie et al., 2009a). In the present study, we implemented two approaches (i.e., in-series and interstitial approaches), allowing us to compare simulation results between the two methods. Here, we describe the structural arrangement of the fibroblasts and myocytes, leaving the description of electrical coupling to Section 2.3.

### 2.3 Random fibroblast insertion in series

**FIGURE 2:**
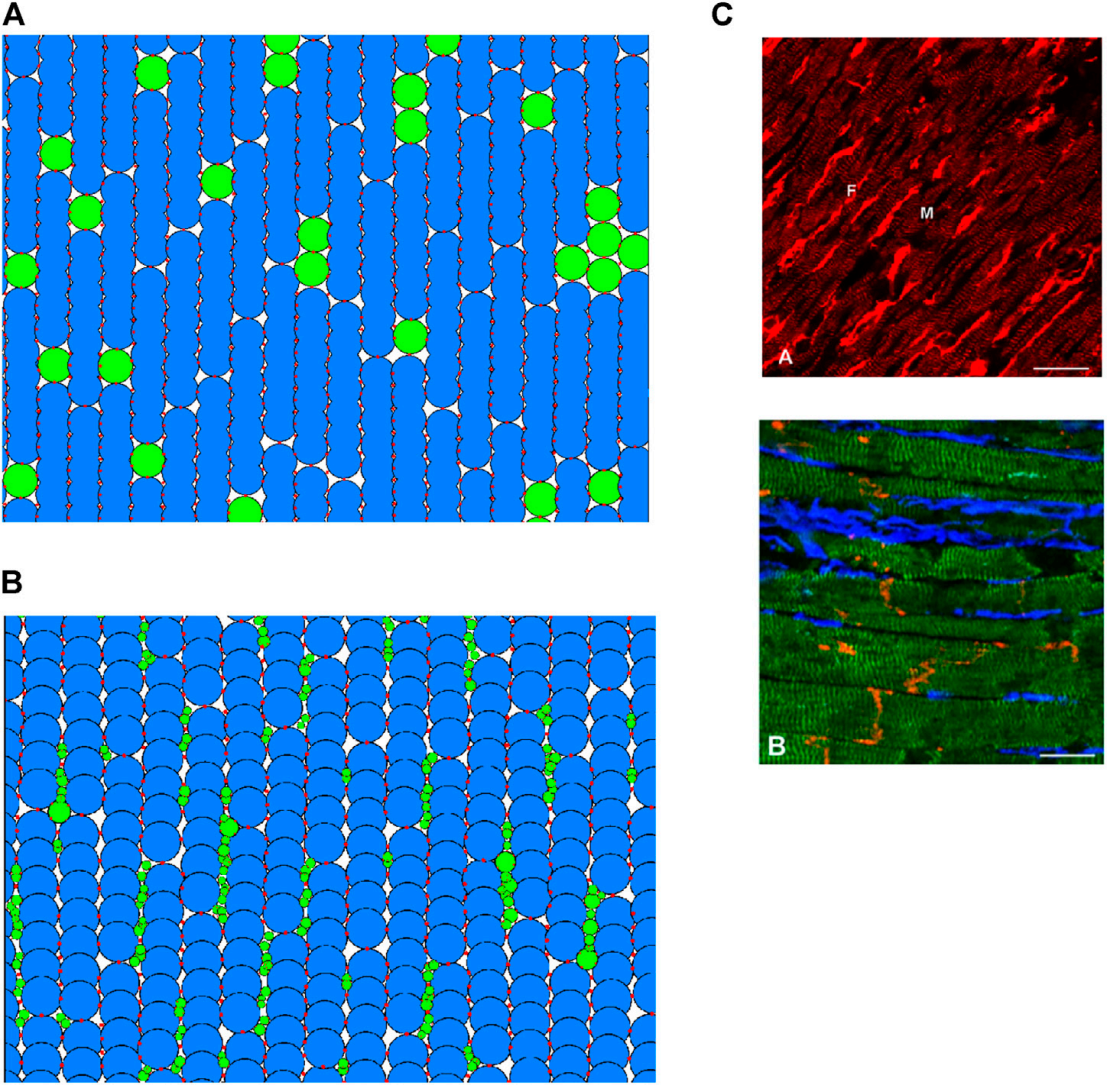
**(A,B)** 2D regions of tissue modelled with DEM using the in-series and interstitial approaches respectively. Blue indicates a myocyte; green indicates a fibroblast; red shows the location of DEM contact bonds. **(C)** Fibroblast organisation from cardiac imaging (Camelliti et al., 2005). (A) Illustration of normal rabbit cardiac tissue—fibroblasts are brightly stained elongated cells (labelled F), myocytes are striated cells (labelled M). (B) Illustration of sheep cardiac tissue—fibroblasts are stained blue, myocytes stained green, connexin43 is red. Scale bars 20 μm.

Previous studies (Sachse et al., 2008; Zlochiver et al., 2008; Xie et al., 2009a) have considered inserting fibroblasts “in-series” with myocytes, which was implemented first in our DEM model. In this case, cells were generated procedurally into columns of fibres, forming a 2D tissue with vertical fibre orientation. As each cell is placed, it has prescribed odds of being a myocyte or fibroblast, according to a desired ratio between the two cell types. In this model, a group of fibroblasts were represented by a single particle of radius *r* = 12.5 μm (assuming to be the same as myocyte particles for simplicity). An example tissue is shown in Figure 2A.

### 2.4 Interstitial fibroblast abundance

Exploiting the versatility of the DEM method, we attempted to construct a coupling approach to represent descriptions and images of fibroblast-myocyte coupling *in-vivo.* According to (Kohl et al., 2005), fibroblasts are arranged in sheets and strands that run in parallel to the prevailing direction of muscle fibres, or bridge gaps between cell groups or layers of myocardium. In essence, every cardiac myocyte is therefore in direct contact to 1 or more fibroblasts *in situ* (Kohl et al., 2005). A scanning electron microscopic studies showed that fibroblasts are spindle-like, slender cells that form an interconnected network of cells that surrounds cardiomyocytes (Rohr, 2012). Imaging study also showed that fibroblasts preferentially reside in the plane of myocardial tissue, along the lateral sides of myocytes rather than between the end-to-end contacts (Xie et al., 2009a). An example of tissue imaging showing the distribution of fibroblasts in cardiac tissue is shown in Figure 2B.

With regards to fibroblast dimensions, freshly-isolated fibroblasts are rounded cells with initial diameters of 7–9 μm, and considering their near-spherical shape, a surface membrane area of 150–250 μm^2^ (Kohl and Gourdie, 2014). However, *in vivo*, the situation is drastically different, where fibroblasts form large sheet-like extensions with irregular folds and extended cytoplasmic processes as described in (Kohl and Gourdie, 2014). A study based on electron microscopy of an individual fibroblast in rabbit sino-atrial node tissue revealed that the total surface area of this cell may be about 1500 μm^2^ or more (Kohl and Gourdie, 2014).

In this study, we attempted to distribute fibroblast and myocyte particles to form a tissue model which is in accordance with the above descriptions and images. The process is somewhat imprecise, as there is no data on the cell size distribution of fibroblasts *in vivo* (Kohl and Gourdie, 2014)*,* and from Figure 2B there appears to be significant variance in fibroblast size. In model development, firstly, strands of myocytes are arranged in vertical fibres. Myocytes are given a slight random rotation between −10 and 10°, and a slight horizontal offset, creating space in between the myocyte particles. This space is filled by randomly generated fibroblast particles of between 1 and 4 μm radius (particles are not generated if they would overlap with existing particles). Fibroblast particle radii are then multiplied by a random factor between 1 and 1.5, to ensure a dense network of physical contacts between particles. Finally, DEM contact bonds are applied following the original DEM atrial tissue model (Brocklehurst et al., 2015; Brocklehurst et al., 2017).

An example of cardiac tissue imaging in Figure 2C shows cluster distribution of fibrosis (fibroblasts). In the model, we took this into account. Fibroblast particles are grouped into individual fibroblast cells by assuming that if two fibroblast particles have a DEM contact bond between them, they are part of the same fibroblast. This particular distribution has a fibroblast-myocyte ratio of 1.27: 1. It is possible to generate tissues of desired F-M ratio by varying the rotational and horizontal offset of the myocytes as well as slightly varying the maximum and minimum particle sizes of the fibroblasts.

### 2.5 Electrical coupling of cells

We assume that all cells in the model, whether myocyte or fibroblast, are electrically coupled with other cells that they are physically connected to. Assuming electrical coupling, thus, electrical excitation waves may propagate throughout the system, despite cell heterogeneity. Handling of electrical wave propagation in the DEM distribution was introduced in (Brocklehurst et al., 2015; Brocklehurst et al., 2017). Individual cells are assumed to be equipotential among their constitute elements, and are considered “neighbours” if there exists a DEM bond between other cells (Brocklehurst et al., 2015; Brocklehurst et al., 2017). The method is adapted to handle arbitrary connections between different numbers of myocytes and fibroblasts.

We introduce a subscript *c* to indicate the type of each cell, *i.e*., *c = m* for myocytes or *c = f* for fibroblasts. The differential equation for the membrane potential of a cell of type *c* is given by:

$$\frac{dV_{c}}{dt}=-\left(\frac{1}{C_{m,c}}\right)\left[I_{c}\left(V_{c},t\right)-I_{ex,c}\left(t\right)-I_{n,c}\left(V_{c},t\right)\right]$$ (1)

where *V*_*c*_ is the membrane potential of the cell; t represents time; *C*_*m,c*_ is the membrane capacitance of the cell; *I*_*c*_ is the ionic membrane current of the cell using the models described in Sections 2.1.1, 2.1.2; *I*_*ex,c*_ is a term representing external stimulus; *I*_*n,c*_ is a contribution from neighbouring cells via intercellular electrical coupling. The external stimulus *I*_*ex,c*_ is defined as (*I*_*ex,m*_ for myocytes; *I*_*ex,f*_ for fibroblasts):

$$I_{ex,m}=\{\begin{array}{c} S_{s,}\;\;\;\;\;\;\;\;\;\;\;\;\;if\;\;\;\;\;\;\;\;S_{t}<t<\;S_{t}+S_{d},\\ 0,\;\;\;\;\;\;\;\;\;\;\;\;\;\;\;\;\;\;\;\;\;\;\;\;\;\;\;\;\;\;\;\;\;\;\;\;\;otherwise,\;\; \end{array}$$ (2)

$$I_{ex,f}=0.$$ (3)

Here, *S*_*s*_ > 0 is the stimulus strength, *S*_*t*_ is the time the stimulus is applied, and *S*_*d*_ is the stimulus duration. The contribution from neighbouring cells *I*_*n,c*_ is given by:

$$I_{n,c}=\overset{n}{\underset{i=1}{\sum }}D^{i}\left(V_{y}^{i}-V_{c}\right),$$ (4)

where n is the total number of neighbouring cells, and $V_{y}^{i}$ is the membrane potential of the $i^{th}$ neighbour, which has cell type y ( y=m or y=f ). The electrical coupling strength (i.e., diffusion coefficients) $D^{i}$ indicates the strength of the gap junction conductance between cells, and is given by:

$$D^{i}=\{\begin{array}{c} D_{m,}\;\;\;\;\;\;\;\;\;\;\;\;\;if\;\;\;\;\;\;\;\;\;y=c=m,\;\\ D_{f}\;\;\;\;\;\;\;\;\;\;\;\;\;\;\;\;\;\;\;\;\;\;\;\;\;\;\;\;otherwise. \end{array}$$ (5)

that is, gap junctions between myocyte-myocyte have conduction strength *D*_*m*_, while gap junctions between myocyte-fibroblast or fibroblast-fibroblast have conduction strength *D*_*f*_. In this study, we use *D*_*m*_ = 1000 ns for myocyte conduction (Xie et al., 2009a; Brocklehurst et al., 2015; Brocklehurst et al., 2017). Following the experimental study of cultured cells in (Rook et al., 1992), we use a range of 0.3 to 8 ns for *D*_*f*_.

### 2.6 Numerical methods

The DEM parameters used in the model are presented in Table 1. Parameters are the same as those used in (Brocklehurst et al., 2015; Brocklehurst et al., 2017), except for the contact stiffnesses, which are modified to account for the altered particle sizes in the present paper.

**TABLE 1:** Default DEM parameter values used in the model.

Parameter	Description	Value
ρ	Density of particles	1.053 g/ml
g	Acceleration due to gravity	$0\;\text{m}/{\text{s}}^{2}$
$k_{n}$	Normal contact stiffness	26 N/m
$k_{s}$	Shear contact stiffness	0.26 N/m
$\beta_{n}$	Normal critical dashpot damping ratio	0.1
$\beta_{s}$	Shear critical dashpot damping ratio	0.1
$T_{F}$	Contact tensile strength	$1\;\times \;10^{200}$
$S_{F}$	Contact shear strength	$1\;\times \;10^{200}$

The computational cycle at each time step is as follows:

• For each cell, loop over neighbouring cells and calculate their contribution to that cell’s electrophysiology, *I*_*n,c*_;
• For each cell, solve the single-cell equations defining electrical and mechanical behaviour of that cell, using the explicit Euler method;
• For each cell, update the particle radii and particle positions, such that the cell length matches the output of the single-cell equations;
• For each contact between particles, solve the force-displacement law, updating the contact forces based on relative particle motion and constitutive contact model; and
• For each particle, solve the law of motion, updating the particle position and velocity due to contact forces.

DEM calculations are performed using Itasca’s PFC version 5. Other calculations are performed using a custom C++ plugin interfacing with PFC. Each step of the computational cycle above must be performed before proceeding to the next step. However, each step is accelerated by parallelising the computation onto the eight threads of an Intel Xeon 3.6 GHz CPU. The DEM equations are solved explicitly by a centred finite-difference scheme. Relatively small timesteps are required owing to the relatively stiff contact springs, and calculation of a minimum timestep is calculated based on spring stiffness and particle radii (Brocklehurst et al., 2015; Brocklehurst et al., 2017). For the in-series and the interstitial model, a timestep of Δ*t* = 0.004 ms and Δ*t* = 0.001 ms is used, respectively. For the coupled single-cell equations, a slightly larger timestep is possible for stable solution, but for simplicity the same timestep as the DEM calculations is used in each case. For the 2D tissue model, it took about 1 hour of desk running time to complete a simulation of 600-ms electro-mechanical activities.

## 3 Results

Using the method described in Section 2 we analysed the effect of FMEC on atrial electrical action potentials and mechanical activities. In simulations, both coupling schemes were considered, i.e., in-series and interstitial, and the results from the two schemes were analysed. Two key parameters are investigated: the gap junction conductance for fibroblast connections *D*_*f*_, as well as the Fibroblast-Myocyte (F-M) ratio, the ratio between the total numbers of each cell type in the tissue.

### 3.1 In-series model

In this section, we analysed the effect of fibroblast insertion in-series to tissue (see Figure 2A). In this model, fibroblasts were inserted between myocytes, breaking the normal end-to-end coupling between myocytes in the fibre direction, affecting electrical propagation.

**FIGURE 3:**
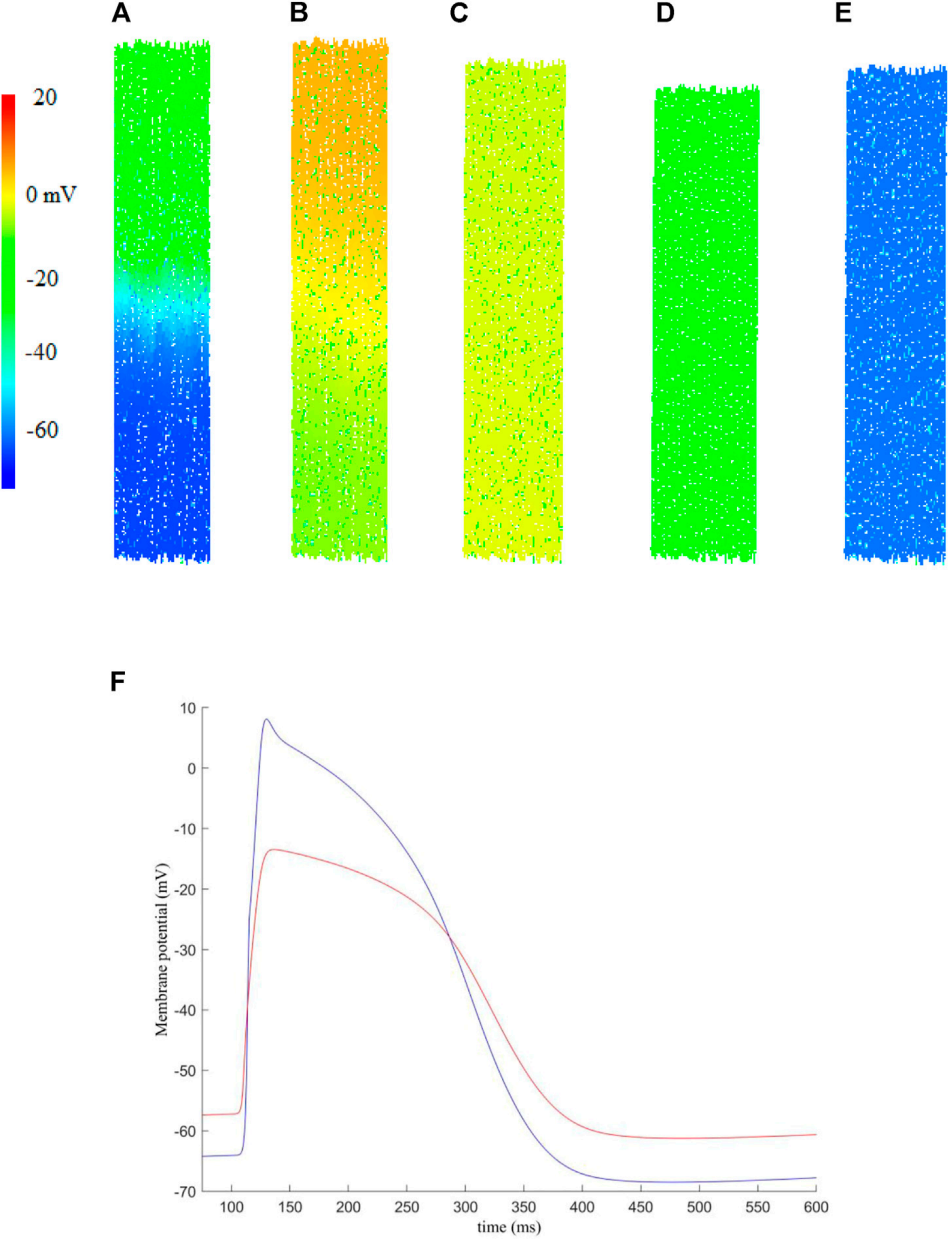
An example of the electro-mechanical activity simulated in a 2D human atrial tissue model including fibroblasts. Snapshots of electrical conduction and tissue contraction were shown at different timings. **(A)** *t* = 108.3 ms, **(B)** *t* = 120.7 ms, **(C)** *t* = 208.3 ms, **(D)** *t* = 208.3 ms, **(E)** *t* = 354.3 ms. **(F)** Membrane potential recorded for a randomly picked myocyte (blue) and fibroblast (red) near the centre of the tissue.

Figure 3 shows simulation results for electrical conduction and mechanical contraction from a 2D DEM model of atrial tissue, with a size of 1.16 mm in width and 6.27 mm in height, fibre orientation of the cells running in the vertical direction. In the tissue model, there are 936 fibroblasts and 2,289 myocytes, giving a F-M ratio of 0.41:1 Here, we used a fibroblast conductance of 0.3 nS. The tissue size though it is small, but sufficient to simulate and visualise the conduction of electrical excitation wave and mechanical contraction.

In simulations, the excitation waves were initiated by a series of external stimuli, which were applied to the upper-most 10% of the tissue at *t* = 100 ms. Each stimulus has strength *S*_*s*_ = 2 nA and duration *S*_*d*_ = 2 ms. Snapshots of electrical wave conduction and tissue contraction are shown in Figures 3A–E with membrane potential of each cell being colour coded.

Snapshots of electrical wave conduction and mechanical contraction/relaxation in the tissue model are presented in Figures 3A–E.

Figure 3A shows the propagation snapshot at *t* = 108.3 ms, when the wavefront passed around the middle of the tissue. At around *t* = 120.7 ms (Figure 3B), the excitation wavefront just passed the bottom edge of the tissue, and the onset of muscle contraction was triggered, which developed further at *t* = 208.3 ms (Figure 3C). At *t* = 280.3 ms, the tissue started to recover from the electrical excitation and full contraction was achieved (Figure 3D). Then the tissue went through relaxation (Figure 3E), completing a full course of electrical excitation and mechanical contraction.

Figure 3F shows representative action potentials recorded from a random myocyte and fibroblast near the centre of the tissue. We note that the fibroblast has a higher resting potential, lower maximum potential and decreased upstroke velocity as compared to those of myocytes, which is in agreement of previous studies [see for example (Camelliti et al., 2005; Maleckar et al., 2009a)].

In order to understand potential impacts of the electrical coupling between myocyte and fibroblasts on atrial electro-mechanical dynamics, further investigations were conducted with varying gap junction conductances between the two cell types and F-M ratio, while the dimension of the tissue remains the same as that in Figure 3. The following quantities are analysed: action potential duration to 90% repolarisation (APD90); the peak potential, or action potential overshoot (APO); the cell’s resting membrane potential (RMP) and the maximum rate of rise of the upstroke of action potential, max. *dV/dt*.

**FIGURE 4:**
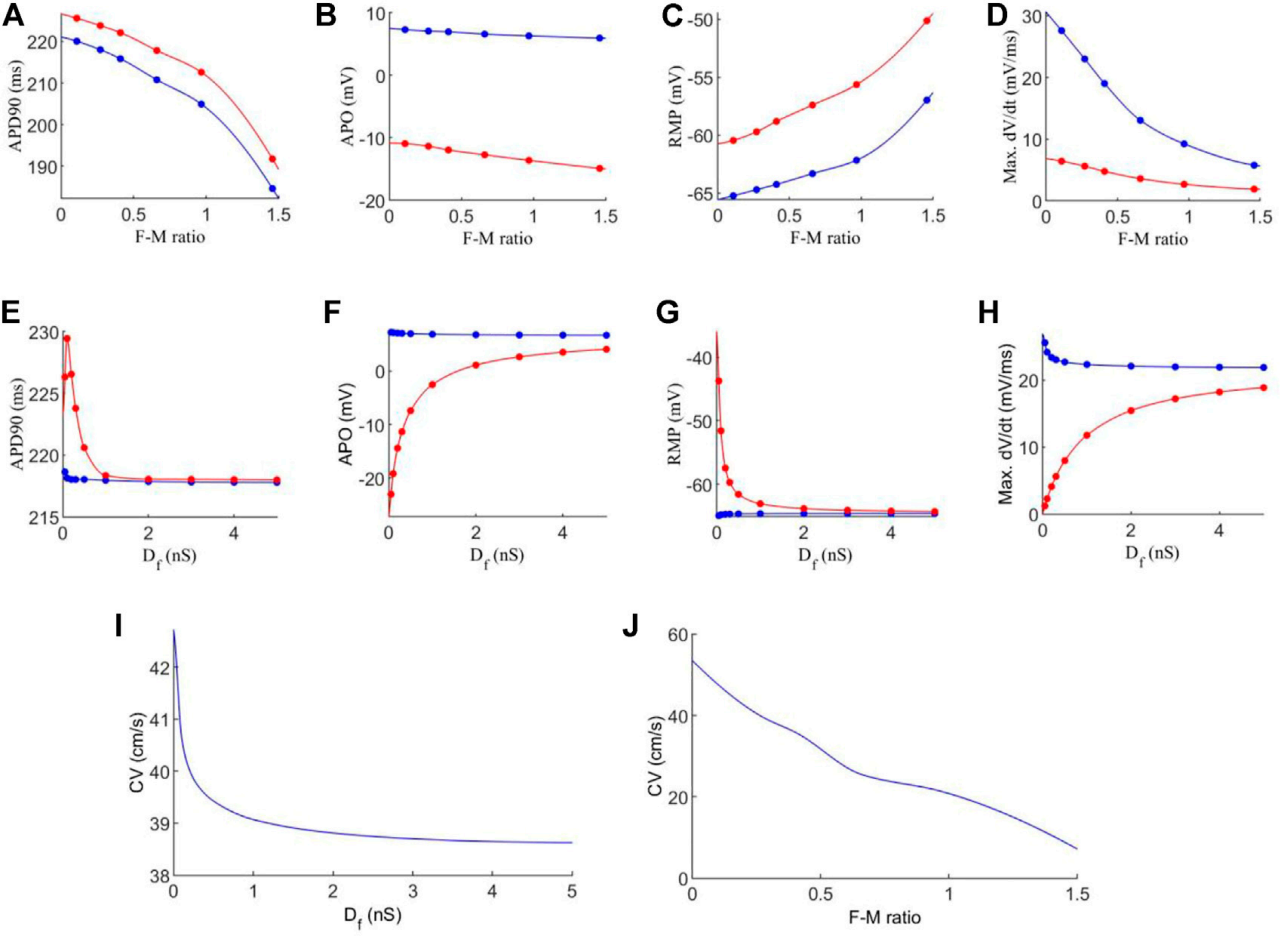
Effects of variations in fibroblast-myocytes coupling strength and F-M ratio on electrical activity of model. For all figures, blue indicates myocytes, and red indicates fibroblasts. Dots indicate a data point. **(A–D)** Characteristics of the action potential with varying F-M ratio (at F-M ratio = 0, the measured values are 225 ms, 9.36 mV, −72.62 and 66.05 mV/ms for APD90, APO, RMP and max. dV/dt respectively for atrial cells). **(E–H)** Characteristics of action potentials with varying *D*_*f*_. **(I–J)** The measured conduction velocity in the fibre direction with varying *D*_*f*_ and F-M ratio respectively. The graph showed F-M ratio maximal up to 1.5 and D_f_ maximal up to 5 nS as beyond these values, the S2 stimulus failed to evoke propagating excitation wave in this setting.

Figure 4 shows the effect of varied coupling strength and F-M ratio on the characteristics of action potentials and conduction velocity of excitation waves. In the simulation, these quantities were calculated for each cell in the tissue, and the mean average was obtained for each cell type in order to account for variations in cell pairings throughout the tissue.

Firstly, we investigate the effects of varying the F-M ratio, while keeping the fibroblast gap junction conductance fixed at *D*_*f*_ = 0.3 nS. The results are shown in Figures 4A–D.

Figure 4A shows a considerable reduction in APD90 with an increase in the F-M ratio, for both fibroblasts and myocytes. In general, fibroblasts have a slightly greater APD90 than myocytes. Fibroblasts begin with an APD90 of 225.6 ms at an F-M ratio of 0.11:1, which decline to an APD90 of 191.8 ms at an F-M ratio of 1.46:1. Myocyte APD90 drops from 220.1 ms to 184.6 ms in the same range. Both curves are similar in trend.

Increase in the F-M ratio also reduces the overshoot of the action potential as shown in Figure 4B. Over the range of the F-M values considered, myocytes have a higher peak potential than fibroblasts. Myocyte APO is less affected by changing the F-M ratio as compared to the fibroblast (changing from 7.3 to 5.9 mV for myocytes versus from −10.9 to −14.9 mV for fibroblasts with the whole range of different values of F-M considered).

Figure 4C shows the effects of varying the F-M ratio on the RMP. As the F-M ratio increases, there is an increase in RMP for myocytes (the RMP changes from −65.2 mV to −57 mV for the whole range of the F-M ratio considered) and fibroblasts (the RMP changes ranges from −60.5 to −50.1 for the whole F-M ration considered).

However, the curves for max. *dV/dt* (Figure 4D) have different trends for myocytes and fibroblasts. Myocytes experience a steeper drop off, from 27.6 to 5.8 mV/ms. Fibroblasts steadily decline from 6.4 to 1.9 mV/ms.

Effect of varying the fibroblast conductance *D*_*f*_ on the atrial electrical activity is shown in Figures 4E–H. Here, the F-M ratio is fixed at 0.27:1. Figure 4E shows the change of APD90 against *D*_*f*_. For fibroblast, the measured APD shows a bi-phasic change with *D*_*f*_. For *D*_*f*_ being increased from *D*_*f*_ = 0.05 nS to *D*_*f*_ = 0.1 nS the measured APD90 first increases from 226.3 ms to 229.4 ms. As *D*_*f*_ increases further, APD90 sharply declines, tending to a final value of 218 ms for *D*_*f*_ = 5 nS. For myocytes, there is only a small variation of 0.84 ms in APD90 for all values of *D*_*f*_ . For both myocytes and fibroblasts, there is little variation in APD90 once *D*_*f*_ increases beyond 2 nS, and at this point the APD90 is very similar for each cell type.

Similarly, myocyte APO (Figure 4F) is unaffected by changes in fibroblast gap junction conductance, remaining close to 7 mV. Fibroblast APO, however, increases sharply from −23 to 4.1 mV as *D*_*f*_ increases from 0.05 to 5 nS. For RMP (Figure 4G), *D*_*f*_ proves again ineffective in modifying myocyte morphology, as RMP remains close to −65 mV. In contrast, fibroblast RMP steeply declines from −43.7 to −64.3 mV. Similar behaviour to the APD90 curve here is that there is little variation in RMP once *D*_*f*_ increases beyond *2nS*, and both cell types are close in RMP at this point. However, changes in *D*_*f*_ do influence max. *dV/dt* values for myocytes (Figure 4H), as we see a variance of 25.6 to 21.9 mV/ms over the range of *D*_*f*_ = 0.05−5nS Over this range, the average fibroblast max. *dV/dt* increases steeply from 1.2 to 18.9 mV/ms.

Finally, the conduction velocity is measured in the tissue along the fibre direction, and the results are shown in Figures 4I–J. We see that increasing *D*_*f*_ and the F-M ratio result in reduction of CV. However, the F-M ratio has a much drastic effect, reducing CV from 47 to 8.5 cm/s over the range of the F-M ratio considered.

Further analyses are performed to quantitatively evaluate the mechanical contraction produced by the tissue, using our electro-mechanical DEM model. The average strain-rate tensor of the tissue is estimated using a best-fit procedure which minimises the error between the predicted and measured velocities of all particles in the tissue. Strain increments can then be accumulated at each time-step throughout the computational cycle, giving curves for the approximate average strains in the tissue. The equations to calculate the strain-rate tensor are described in (Itasca Consulting Group Inc., particle flow code version five user manual, Minneapolis, MN 2014).

**FIGURE 5:**
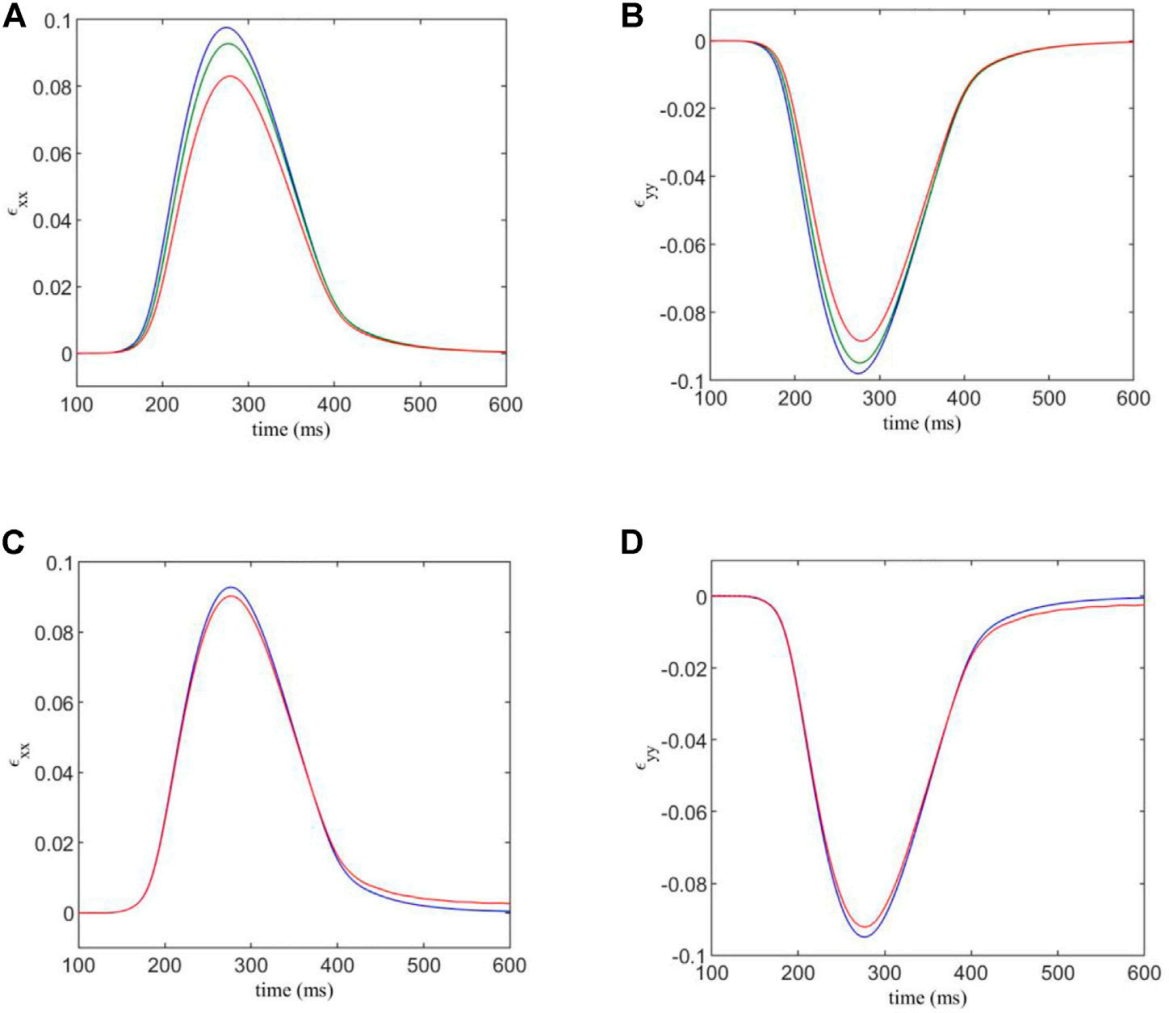
**(A,B)** components *ɛ*_*xx*_ and *ɛ*_*yy*_ of the estimated strain tensor, versus time. The F-M ratios are given by: 0.108:1 (blue); 0.409:1 (green); 0.967:1 (red). **(C,D)** components *ɛ*_*xx*_ and *ɛ*_*yy*_ of the estimated strain tensor, for varied normal and shear contact stiffnesses versus time. Blue indicates the strain curve for standard contact stiffnesses, and red indicates the strain curve for the factor 1.5 modification for contacts involving fibroblasts.

Firstly, we analysed the average strain while varying the fibroblast gap junction conductance *D*_*f*_, but this parameter was found to have almost no effect on the strain in the tissue. However, the F-M ratio does affect the strains, as shown in Figures 5A,B. Here, a similar sized tissue to that above was stimulated at *t* = 100 ms, and *D*_*f*_ = 0.3 nS.

We see that for an F-M ratio of 0.108:1, for the *xx* -component of the strain tensor, there is a maximum value of 0.0977, while for a F-M ratio of 0.967:1, the strain peak is at 0.083. This corresponds to a substantial 14% reduction in strain in the tissue. Results for the *yy* -component are similar, as the strain varies from −0.098 to −0.0885, corresponding to a 10% reduction in tensile strain.

Using the DEM model, it is possible to increase the strength of DEM contacts involving a fibroblast, in order to simulate the mechanical stiffening often associated with fibrosis. We multiply the normal and shear contact strengths, *k*_*n*_ and *k*_*s*_, by a factor of 1.5 when either end of a contact is part of a fibroblast. The results are shown in Figures 5C,D. Here, *D*_*f*_ = 0.3 nS and the F-M ratio is 0.409:1.

We see that there is only a slight difference in maximum strain for each case. This is somewhat expected, as the majority of the strain in the system is generated by the myocyte contraction, which is unaffected by the changes in DEM contact stiffness of fibroblasts. However, there is still a 2.7% variation in maximum strain component *ɛ*_*xx*_ between the two cases, and a 2.9% variation for *ɛ*_*yy*_ due to the mechanical stiffening caused by fibrosis.

### 3.2 Interstitial model

**FIGURE 6:**
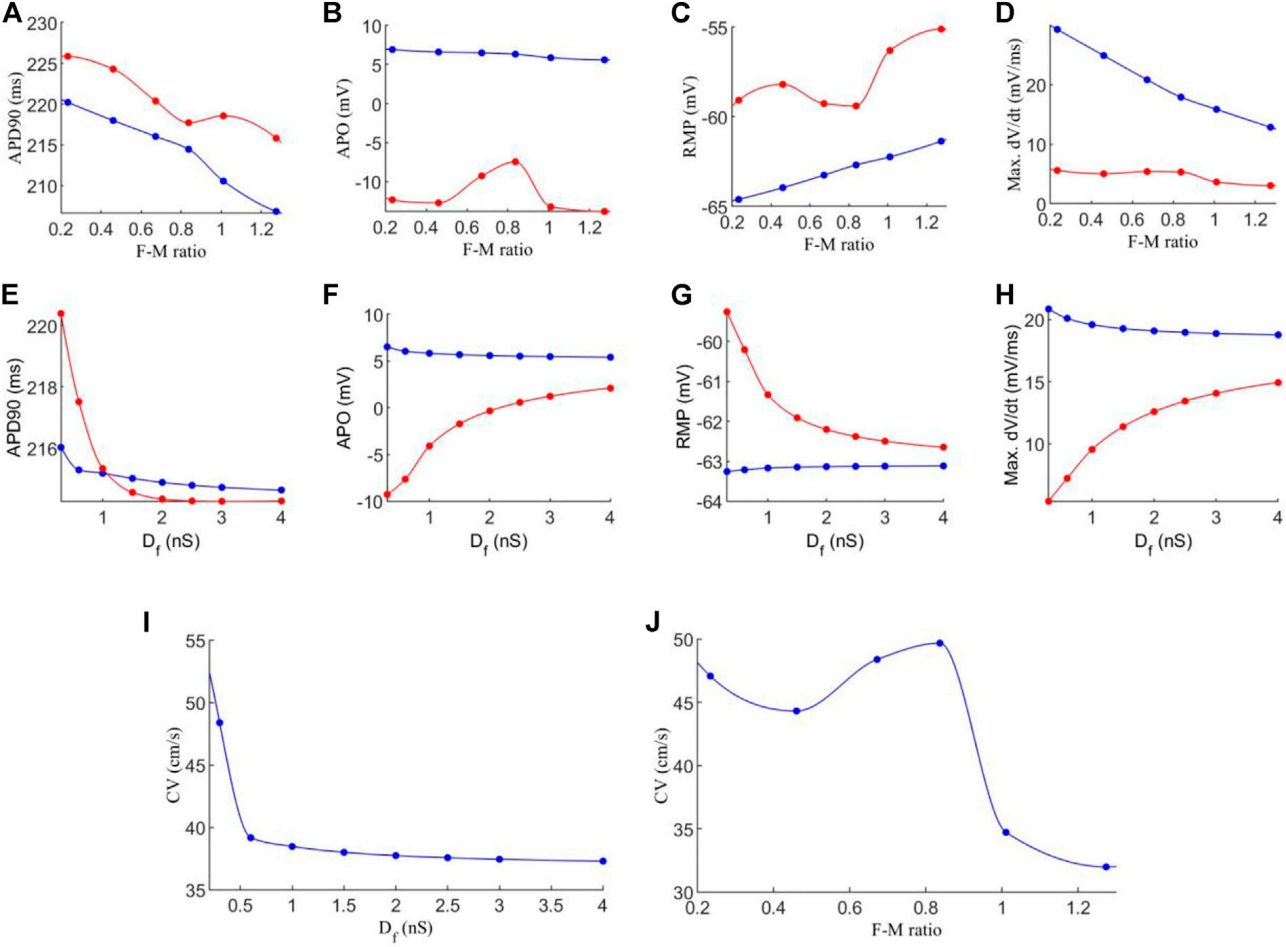
Effects of varying F-M ratio and electrical coupling strength between fibroblast-myocytes on the characteristics of action potential and excitation wave conduction in the interstitial model. For all figures, blue indicates myocytes, and red indicates fibroblasts. Dots indicate a data point. **(A–D)** Characteristics of action potentials with varying the F-M ratio. **(E–H)** Characteristics action potential with varying *D*_*f*_. **(I,J)** Measured conduction velocity in the fibre direction with varying *D*_*f*_ **(I)** and F-M **(J)** respectively. The graph showed F-M ratio maximal up to 1.25 and *D*_*f*_ maximal up to 4 nS as beyond these values the S2 stimulus failed to evoke propagating excitation wave in this setting.

The analysis of Section 3.1 is repeated using the interstitial model (see Figure 2B). Myocyte coupling in the fibre direction is intact, but coupled active fibroblasts in the laminar clefts of the myocyte fibres is still expected to affect electrophysiology in the tissue. As before, computations are conducted on a tissue which is close to 1.16 mm wide and 6.27 mm high (varying depending on the exact number of fibroblasts). We first analyse the APD90, APO, RMP and max. *dV/dt*, as shown in Figures 6A–H. As before, the quantities shown are the mean average for all cells across the tissue.

Figure 6A shows the measured APD90 against the F-M ratio. As F-M increases, there is an APD90 reduction for both myocytes and fibroblasts, as before. However, the effect is less severe than that for the in-series model, as here the APD90 does not fall below 206 ms for myocytes and 215 ms for fibroblasts given the whole range of F-M ratio considered. For APO (Figure 6B), myocytes experience only a slight reduction as the number of fibroblasts increases, and the values are very similar to for the in-series model. However, fibroblast APO slightly increases to −7.5 mV as F-M increases, before falling to −14 mV. For RMP (Figure 6C), myocytes appear unaffected by the coupling methods, as the myocyte RMP’s are quantitatively similar to the equivalent in-series values. However, the fibroblast curve presents different morphology to the in-series model, reaching a minimum RMP value of −59.4 mV at an F-M ratio of 0.84:1. For max. *dV/dt* (Figure 6D), the in-series and interstitial models are quantitatively and qualitatively very similar under variance of F-M.

When analysing the effect of varying *D*_*f*_, we see that for APD90 (Figure 6E) the results are similar to the in-series model, though in this case for fibroblasts falls to a lower value of 214.5 ms. For APO (Figure 6F) the myocyte values are again similar to the in-series model, but the fibroblasts APO is higher for low values of *D*_*f*_. For RMP (Figure 6G), the myocyte values are unaffected by the coupling method. For fibroblasts, at *D*_*f*_ = 0.3 nS, in the interstitial model there is an RMP of −59 mV compared to −43 mV in the in-series model. As *D*_*f*_ increases, the results become more similar. For max. *dV/dt* (Figure 6H), the trends are the same as for the in-series model, but the myocyte max. *dV/dt* is slightly lower, and the fibroblast max. *dV/dt* varies over a smaller range of 5 to 15 mV/ms. For CV (Figure 6I), when increasing *D*_*f*_, CV is initially higher than for the in-series model, at 48.5 cm/s here, but after *D*_*f*_ = 0.6 nS the two models produce very similar values. However, the curve for varying F-M ratio (Figure 6J) again presents different morphology here, actually reaching a peak value of 49.6 cm/s at an F-M ratio of 0.84:1 before declining to 32 cm/s over the range considered.

**FIGURE 7:**
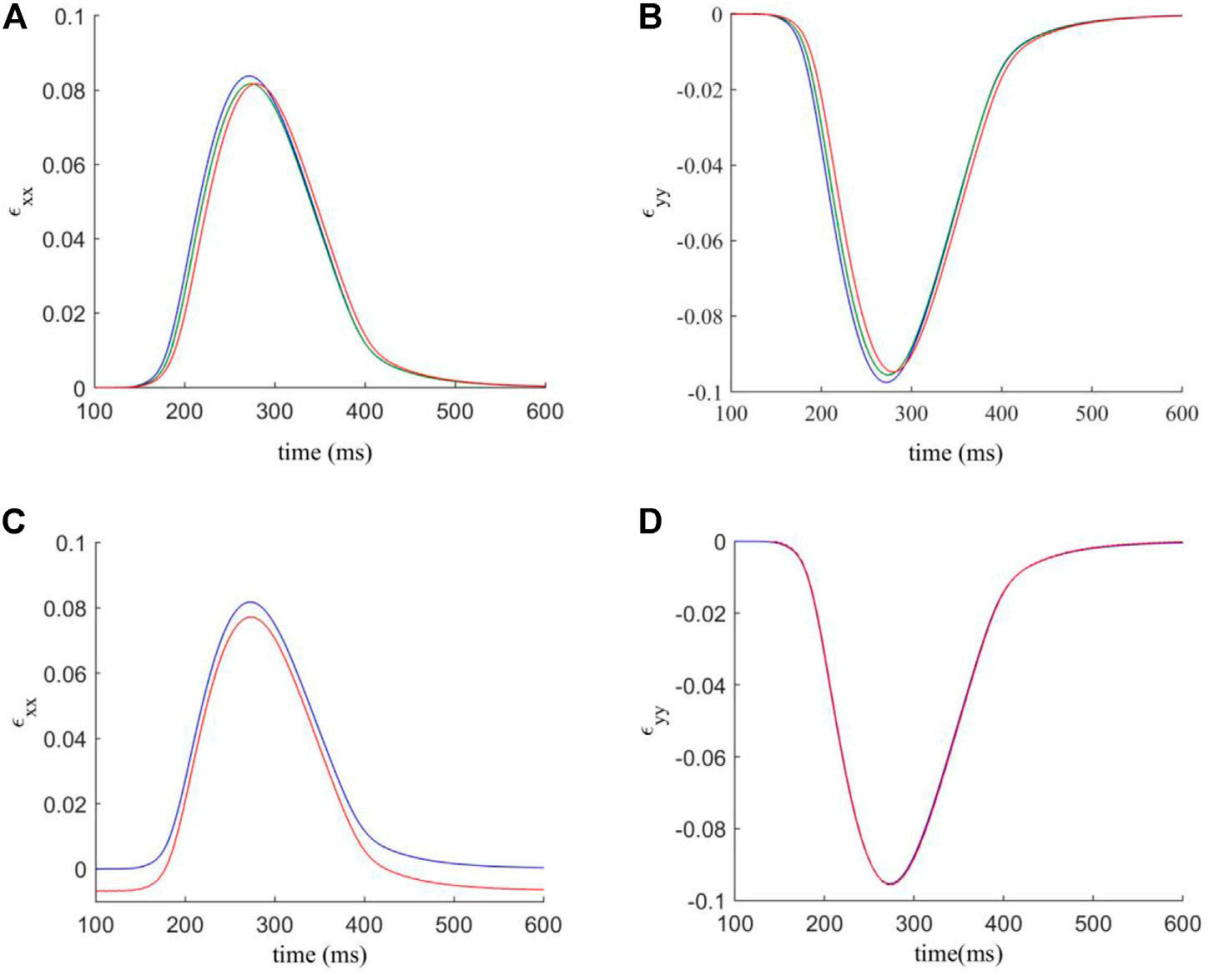
**(A,B)** components *ɛ*_*xx*_ and *ɛ*_*yy*_ of the estimated strain tensor, versus time. The F-M ratios are given by: 0.24: 1 (blue); 0.84: 1 (green); 1.27: 1 (red). **(C,D)** components *ɛ*_*xx*_ and *ɛ*_*yy*_ of the estimated strain tensor, for varied normal and shear contact stifnesses versus time. Blue indicates the strain curve for standard contact stiffnesses, and red indicates the strain curve for the factor 1.5 modification for contacts involving fibroblasts.

The analysis of the strain in the tissue is repeated as for the in-series model (Figure 5). Figures 7A,B analyses the average strain in the tissue against time. In general, the interstitial model has lower values of *ɛ*_*xx*_ and less variation in response to F-M ratio changes (peaks ranging from 0.084 to 0.0815). Values of *ɛ*_*yy*_ are similar though with again less affected by changes in the F-M ratio. In Figures 7C–D the contacts involving fibroblasts are again stiffened by a factor of 1.5. When stiffened, interstitial coupling led to *ɛ*_*xx*_ having a resting value of −0.007 and not zero. Also for *ɛ*_*xx*_ there is a significant reduction of roughly 10% in maximum strain when compared to the interstitial model. For *ɛ*_*yy*_ the results are similar in both modelling approaches, though for the interstitial model there is no change after stiffening.

### 3.3 Plane wave propagation

**FIGURE 8:**
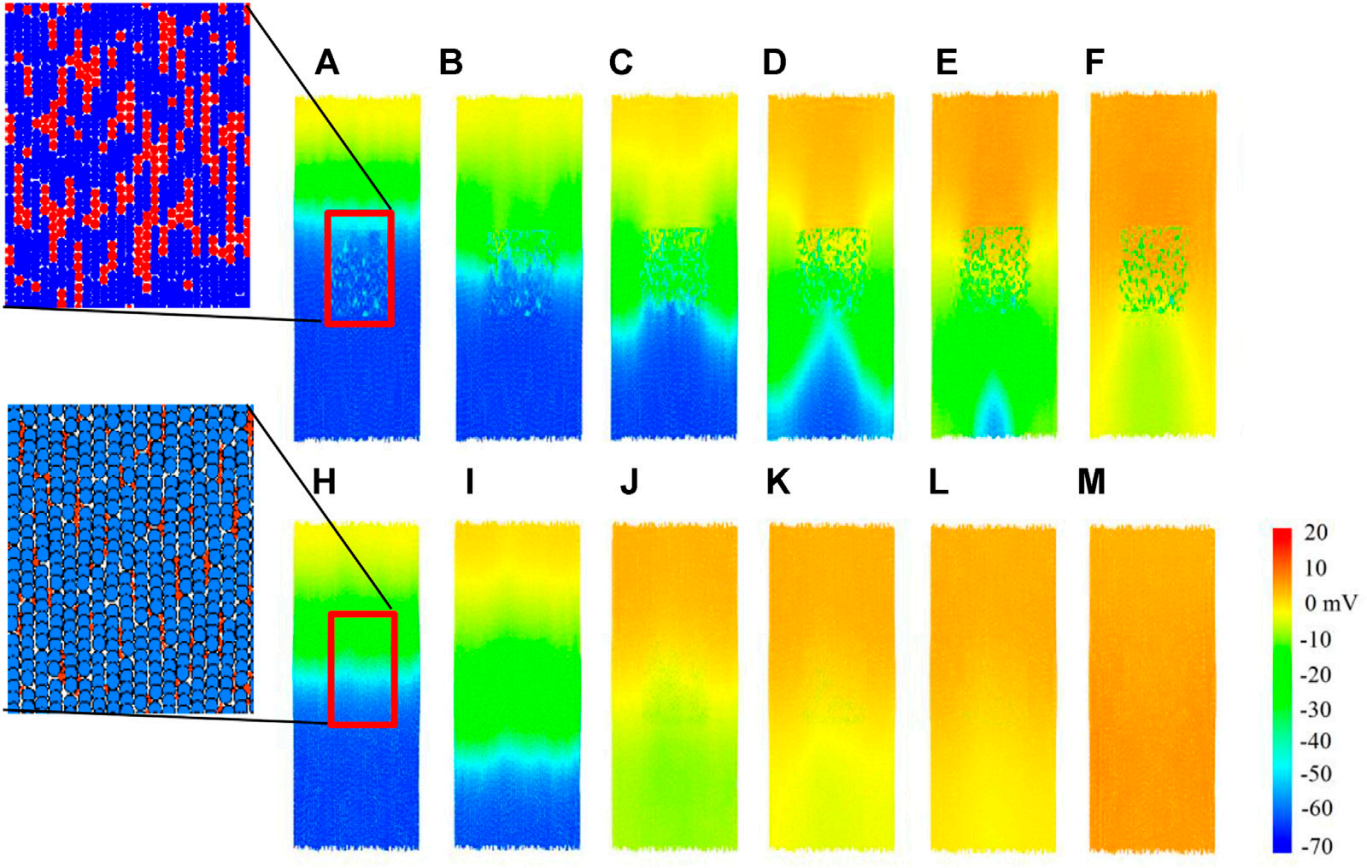
Plane wave propagation past a central fibrous region. Cells are coloured by their membrane potential as shown in the colour bar. Inset: spatial configurations of fibroblasts in the corresponding in-series and interstitial models. **(A–F)** Snapshot of excitation wave conduction in the in-series model at *t* = 102.7, 104.7, 107.1, 109.9, 116.3, 122.3 ms respectively. **(H–M)** Snapshot of excitation wave conduction in the interstitial model at *t* = 104.4, 108.0, 114.3, 116.1, 117.0, 120.6 ms respectively. In both cases, representative snapshots at different timings were chosen for illustration purposes.

In this section, we compare the impact of the two modelling approaches on the plane wave propagation past a central fibrous region. A tissue of size 2.32 mm wide and 6.27 mm high is constructed for each coupling method, with a central rectangular region containing ∼930 fibroblasts in each case. A stimulus is applied from the top of the tissue, and the results are shown in Figure 8; and the corresponding time courses of atrial electromechanical activities are shown in Supplementary Videos S1, S2. In both cases, results for *D*_*f*_ = 0.3 nS were shown for illustration to show how the fibroblast affect the conduction pattern of excitation waves.

For the in-series model, the top row Figures 8A–F, we see that the central fibrous region has a blocking effect on the plane wave propagation, producing altered wave patterns in the lower region of tissue. However, for the interstitial model in Figures 8H–M, plane wave propagation was unaffected by the central fibrous region. Only slight curvature below the fibrous region is visible, in the range of −10 to 10mV. To see how the fibroblast cluster affects the rate-dependent conduction of excitation wave, for both models, a second premature stimulus after 350 ms was introduced (not shown), but the wave patterns were the same as for the first stimulus in both cases at this stimulus time interval. The observed effect of conduction blocking in the in-series model may predispose to initiation of re-entry, leading to onset of atrial re-entrant arrhythmia; but the tissue is too small and the conduction too fast to accommodate it, and is beyond the scope of this study.

## 4 Discussion

In this study, we developed a 2D DEM-based electro-mechanical model of human atrial tissue by using the discrete element method approach. Using the model, we investigated possible functional impacts of FMEC on the electrical and mechanical activities of the human atrial tissue. Our major findings are: 1) the FMEC impairs the electrical and mechanical activities of the cardiac tissue, which are manifested by abbreviated action potential duration, reduced conduction velocity of excitation waves and tissue strain during contraction, suggesting impaired muscle contractility; 2) the observed effects of FMEC are dependent on their population and the coupling strength. With an increase in the F-M ratio or the coupling strength, the observed APD abbreviation, reduction in the conduction velocity of excitation waves and product of muscle strain were further decreased; and 3) the observed effects of fibroblasts rely on how the fibroblasts are distributed and coupled to myocytes. In the case of in-series coupling, there was a monotonical relationship between the measured changes in the electrical activities (i.e., reduction of action potential duration and conduction velocity) and the increase of F-M ratio or the coupling strength. However, in the case of interstitial coupling, such relationship became complex and multi-phasic as the measured conduction velocity of excitation was either decreased or increased depending on the F-M ratio. In terms of mechanical contraction, the in-series coupling caused more reduction in the measured strain than the interstitials coupling. Collectively, these findings provide new insights into the functional impacts of FMEC on impairing cardiac electrical and mechanical functions.

### 4.1 Effects on electrical activity

Due to differences in the membrane of ion channel kinetics and properties, fibroblasts present different resting membrane potential and cellular excitability to myocytes (Kohl et al., 2005; MacCannell et al., 2007; Nattel, 2018). As such, it is believed that fibroblasts may modulate the electrical activity of myocytes via electrical coupling arising from connexin expression between the two (Aguilar et al., 2014; Ongstad and Kohl, 2016; Kucera et al., 2017; Nagaraju et al., 2019), contributing to increased risks of arrhythmogenesis (Xie et al., 2009b; Xie et al., 2009c; Nguyen et al., 2012; Thomsen and Calloe, 2016) and instability of re-entrant excitation (Nayak et al., 2013; Zimik and Pandit, 2016). In this study, we observed that at the single cell, the FMEC abbreviated action potential duration, reduced the overshoot of action, elevated resting membrane potential, which can be attributable to the difference in the resting membrane potentials between fibroblasts and myocytes, as well as the less excitability of fibroblast. Due to a more depolarised resting potential and less excitability of fibroblast, the interaction of the fibroblast to the myocyte can be regarded as bi-phasic during a full time course of excitation: a driver when its membrane potential is higher than that of myocytes, and a load when its membrane potential is lower than that of myocytes. When myocyte and fibroblast are quiescent, the myocyte-fibroblast coupling produced a depolarising current flowing from the fibroblast to myocytes causing its resting potential to be elevated. Upon stimulations, the membrane potential of myocytes excurses a rapid change producing overshoot of action potential, which is higher than that of fibroblast. In this case, the myocyte-fibroblast coupling produced a dynamical repolarising current flowing from the fibroblast to the myocytes, causing reduced overshoot and action potential duration of myocytes (Xie et al., 2009a). At the tissue level, such modulation actions of fibroblast to myocytes were reflected by reduced conduction velocity of excitation waves. Our simulation results are in agreement with previous computational modelling studies (Gaudesius et al., 2003; Everett and Olgin, 2007; MacCannell et al., 2007; Miragoli et al., 2007; Sachse et al., 2008; Maleckar et al., 2009; Xie et al., 2009; Zhan et al., 2014), mounting evidence in showing the impairing effects of FMEC on cardiac electrical activity. Note that, we observed that with the FMEC coupling and an increase in the F-M ratio, the APD90 of atrial myocytes was abbreviated. Such observation of abbreviated APD90 *via* FMEC coupling was consistent to the observation of (Maleckar et al., 2009a), and the study of Sachse et al. (2008) in the case of a large electrical coupling resistance (10 GΩ). Such APD90 abbreviation may be attributable to the reduced activation of the L-type Ca^2+^ channel arising from a reduced overshot of the action potentials, which were observed in previous studies (MacCannell et al., 2007; Sachse et al., 2008; Maleckar et al., 2009b; Xie et al., 2009).

The distribution of fibroblast in cardiac tissue is complex, both in-series and interstitial distribution. The developed DEM based 2D cardiac tissue model allowed us to investigate the modulation of fibroblast to cardiac excitation wave conduction in both in-series and interstitial coupling scenarios. Our results showed that while both coupling schemes showed similar actions of fibroblast on cardiac excitation and conduction, there is a subtle difference in the measured conduction velocity when the F-M ratio is altered. In the case of in-series coupling, increase of the F-M ratio or the coupling strength produced a monotonic decrease in the conduction velocity. This is attributable to the fact that the fibroblasts are distributed in the principal conduction pathway along the tissue fibre, and form a lower excitable gap between adjacent myocytes, impeding the conduction of excitation waves. However, in the case of interstitial coupling, though the increase in the FMEC caused a monotonic decrease of conduction velocity, following the similar trend as in the in-series coupling, the increase in the fibroblast population (F-M ratio) produced a complex pattern. In simulations, when the F-M ratio was increased, the measured conduction velocity first decreased when F-M ratio was below 0.5, however, it then increased when F-M ratio was between 0.5–0.9 and then decreased again at F-M ratio greater than 0.9. Such complex multi-phasic changes in the measured conduction velocity may be attributable to the balance of a driver or a load the fibroblasts act on myocytes functionally. As the fibroblasts are distributed at the side of myocytes always of the principal conduction pathway along the cardiac fibres, their actions to modulate cardiac excitation wave conduction rely on how they modulate the maximal upstroke velocity of action potentials and cellular excitability of cardiac cells. When the population of fibroblast is low (<0.5), the fibroblast acted more like an electrotonic load, which reduced the maximal upstroke velocity of the action potential, leading to a reduced conduction velocity. When the F-M ratio was increased, in one way the coupling reduced the maximal upstroke velocity that would lead to reduction of the conduction velocity, and in the other way it acted as a driver, which elevated the resting potential of myocytes that would increase cellular excitability leading to an increased conduction velocity. These two actions compete, and at the range of F-M ratio between 0.5 and 0.9, the driver action of the fibroblast dominated resulting in an increased conduction velocity. When F-M ratio is further increased (>0.9), the load action dominated leading a decrease in the conduction velocity again.

### 4.2 Effects on mechanical activity

Cardiac diseases such as myocardial infarction with increased fibrosis population are often accompanied by contractile dysfunction of the heart, which may result in cardiovascular mortality (Naccarella et al., 2000). However, few studies have attempted to investigate the functional impacts of myocyte-fibroblast coupling on the mechanical activities of cardiac tissue and most previous studies have focused on the investigation of the electrical consequence of the coupling. In their study, Bazhutina et al. (2021a), Bazutina et al. (2021b) investigated at the single cell level how the electrotonic interaction with fibroblasts affects the mechanical activity of cardiomyocyte, resulting in reduction of contractility manifested by reduced cell length shortening and the product of active force. These compromising effects of FMEC on cardiac electromechanics were enhanced by increasing number of coupled fibroblasts. At the tissue level, Zhan and Xia (2013) developed a 2D electro-mechanical model of human atrium based on the finite element method. Their simulation results showed that the myocyte-fibroblast coupling decreased strains in fibrotic tissue. In the present study, we developed a 2D model of atrial electro-mechanics based on discrete element method with consideration of the discrete nature of cardiac tissue and fibroblasts, enabling us investigating not only electrical coupling and changes of F-M ratio, but also the in-series or interstitial coupling between fibroblasts and myocytes on electro-mechanics of cardiac tissue. Our simulation results showed that the FMEC impaired tissue’s contractile activity. We went further to demonstrate that such compromising effects of FMEC was dependent on the way how fibroblast were distributed in cardiac tissue. Whilst both in-series and interstitial coupling schemes impaired cardiac tissue contractility resulting in a reduced strain, the effect of in-series coupling is more dramatical than that of the interstitials coupling. The observed comprising effects of FMEC on cardiac mechanical activity was enhanced by the increase in the number of fibroblasts or the strength of the coupling, providing insights into understanding of how the increased fibrosis (fibroblasts) can cause dysfunctional contractility of the heart in many cardiac diseases such as myocardial infarction.

### 4.3 Limitation of the study

The present model has several limitations which may be improved in later model development. The single cell electro-mechanical model of the human atrial cell implemented the Courtemanche et al. (1998) and Rice et al. (2008) models for cardiac electrophysiology and filament kinetics. The limitations of both models were well documented. The fibroblast single-cell model was not verified against human experimental data yet (MacCannell et al., 2007; Maleckar et al., 2009a). The 2D tissue model developed in this study was based on an idealised tissue geometry and fibre orientation, did not consider 3D anatomical structure of atrial tissue and potential regional difference in cellular electrophysiology and possible atrial fibrillation-induced electrical remodelling (Colman et al., 2013). However, the development of complete electromechanical single-cell models for various regions of the human atria and fibroblast warrants further study as more experimental data becomes available. As the DEM model takes each myocyte as a 7-element assembly with each element representing a spatial resolution of about 20 μm, it is expected a high demand for super-computing power for realistic or patient-specific 3D whole atria model simulations. In the model development, we used an idealised cellular geometrical structure, without considering the specific features of atrial cell morphology that may differ to ventricular myocytes. Therefore, the model warrants further development to incorporate atrial specific geometrical structures. The present study implemented simple isotropic electrical coupling without considering anisotropic features of cardiac tissue, also simply took the fibroblast as electrically active but mechanically passive. Such simple assumptions require to be addressed when more experimental data on the mechanical properties are available. In addition, feedback from mechanical contraction to the electrical formulation was not considered here yet, which can be addressed by introducing a stretch-activated currents. In simulations, we only investigated potential impacts of altered configurations of fibroblast distributions on atrial electro-mechanical dynamics, and analysed changes of averaged characteristics of action potentials and conduction of excitation waves from cells throughout the tissue, without considering possible effects of altered spatial configurations of myocytes, or analysis of variability of those characteristics. Investigations on altered myocytes arrangements, combined variations of myocytes and fibroblasts spatial configurations, different atrial cell models such as the Maleckar et al. model (2009) on the characteristics of atrial electrical and mechanical activities and their variability warrant further studies. Though we made it clear about potential limitations of the present model, which warrant further development, however, these limitations do not affect our conclusions on how FMEC impairs electro-mechanical functions of cardiac tissue.

### 4.4 Potential implication of the study

Atrial fibrillation is a most common cardiac diseases, impairing the ability of the heart to pump blood *via* mechanical contraction. Whilst mechanisms underlying the impaired mechanical function may be related to ion channel and tissue structural remodelling, it is also possibly related to the increased fibrosis/fibroblasts (Kohl and Gourdie, 2014; Nattel, 2018). In this study, our simulation results showed that FMEC impaired atrial electrical and mechanical functions. Further experimental and clinical studies are warranted to analyse possible effects of targeting the FMEC on compromising the electrical and mechanical functions of the heart, in order to evaluate whether they constitute valuable approaches for new treatments of atrial fibrillation.

### 4.5 Conclusion

In this study, we developed a 2D tissue model of electromechanical dynamics of atrial tissue using the Discrete Element Method by taking into considerations the discrete nature of myocytes and fibroblast. Our simulation results show that the FMEC impairs tissue’s electrical and mechanical function, which is enhanced by the increased F-M ratio or the coupling strength. Such compromising effects of the FMEC were also dependent on the way how fibroblasts are distributed among cardiac cells, with in-series coupling showing more impacts than the interstitial coupling. These findings provide new insights into understandings of how the increase of fibroblasts impairs atrial electrical conduction and mechanical contractility.

## Data Availability

The original contributions presented in the study are included in the article/Supplementary Material, further inquiries can be directed to the corresponding authors.
